# Functional brain changes after alternative pharmacological interventions in posttraumatic stress disorder: A systematic review of clinical trials

**DOI:** 10.1002/brb3.3292

**Published:** 2023-10-20

**Authors:** Shahab Lotfinia, Amin Afshar, Aram Yaseri, Miranda Olff, Yann Quidé

**Affiliations:** ^1^ Department of Clinical Psychology, School of Medicine Shahid Beheshti University of Medical Science Tehran Iran; ^2^ Faculty of Medicine Qazvin University of Medical Science Qazvin Iran; ^3^ School of Medicine Shahid Beheshti University of Medical Science Tehran Iran; ^4^ Department of Psychiatry Amsterdam University Medical Centers Location AMC, Amsterdam Public Health Amsterdam The Netherlands; ^5^ ARQ National Psychotrauma Centre Diemen The Netherlands; ^6^ NeuroRecovery Research Hub, School of Psychology The University of New South Wales (UNSW) Sydney Sydney New South Wales Australia; ^7^ Neuroscience Research Australia Randwick New South Wales Australia

**Keywords:** functional magnetic resonance imaging, hydrocortisone, oxytocin, posttraumatic stress disorder

## Abstract

**Background:**

Posttraumatic stress disorder (PTSD) is a complex and heterogeneous mental health condition that can develop after exposure to a traumatic event. Clinical trials have used alternative pharmacological agents to treat PTSD, but their associated neural correlates remain unclear. The present systematic review aims to summarize the changes in brain function associated with the use of these alternative pharmacological agents in PTSD.

**Methods:**

Clinical trials using functional magnetic resonance imaging, either at rest or during the performance of tasks, were included if they compared the effects of alternative pharmacological agents between PTSD patients and either trauma‐exposed controls or never‐exposed healthy controls.

**Results:**

Sixteen studies were included, of which 11 used intranasal oxytocin, 2 used hydrocortisone, and 3 used delta‐9‐tetrahydrocannabinol (THC). Oxytocin administration was associated with the normalization of functional connectivity between the ventromedial prefrontal cortex and amygdala as well as enhanced the function of brain regions specifically involved in emotion processing (e.g., amygdala), working memory (e.g., dorsolateral prefrontal cortex), and reward (e.g., putamen). Hydrocortisone did not influence brain function at rest or during the performance of an autobiographical memory task, whereas THC was associated with the reduction of the amygdala and increased medial prefrontal cortex activation.

**Conclusions:**

This systematic review identified preliminary evidence for normalizing brain function after the use of alternative pharmacological agents. Importantly, sex‐specific differences were noted, in particular when using oxytocin, that will require further investigation.

## Graphical abstract

The present systematic review of clinical trials identified preliminary evidence for normalization of brain function following the administration of pharmacological agents, including intranasal oxytocin, hydrocortisone, and delta‐9‐tetrahydrocannabinol. Overall, normalization of the vmPFC–amygdala connectivity following oxytocin administration was the most consistent finding, but the interpretations of the results from this systematic review are limited by the relatively small number of clinical trials included.

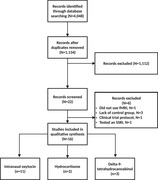

## 1 INTRODUCTION

Posttraumatic stress disorder (PTSD) is a severe psychiatric disorder that can arise after exposure to a traumatic event. Although around 6% of all people exposed to a traumatic event will develop PTSD (Breslau, 2001; Bromet et al., 1998; Mclaughlin et al., 2015), these rates are significantly higher (25%–35%) for assault victims, refugees, or combat veterans (Alexandra Kredlow et al., 2022). Symptoms of PTSD include persistent re‐experiencing of the traumatic event, avoidance of trauma‐associated stimuli, negative thoughts or feelings, and increased arousal that persist for at least a month after trauma exposure (American Psychiatric Association, 2013). Clinical manifestations and symptom intensity of PTSD are very heterogeneous (Regier et al., 2013), possibly implying the involvement of multiple neurobiological systems (Albucher & Liberzon, 2002).

Among the numerous theories proposed to explain PTSD, including the emotional processing to fear model (Foa & Kozak, 1986), the dual‐representation theory (Brewin et al., 1996), or the cognitive model of PTSD (Ehlers & Clark, 2000), most neuroimaging studies have been based on the *failure to inhibit fear* model of PTSD (Liberzon & Sripada, 2007; Rauch et al., 2006). This model implies insufficient top–down regulation from the medial prefrontal cortex (mPFC) to the amygdala (Liberzon & Sripada, 2007; Rauch et al., 2006; Woodward et al., 2006), leading to the hyperactivation of the amygdala. Changes in amygdala–mPFC connectivity can lead to failure to suppress the so‐called default mode network (Greicius & Menon, 2004) during cognitive engagement (Selemon et al., 2019). This aberrant top–down regulation can be normalized following successful psychological and/or pharmacological treatments (Quidé et al., 2012). Anatomical results from the Enhancing NeuroImaging Genetics through Meta‐Analyses consortium indicate that smaller hippocampal and amygdala, in addition to smaller left and right lateral orbitofrontal gyri, were evident in people with current PTSD compared with trauma‐exposed control (TEC) subjects (Logue et al., 2018; Wang et al., 2021), although smaller hippocampal volume may be present prior to trauma exposure and could represent a risk factor to develop PTSD (Gilbertson et al., 2002; Quidé et al., 2018). Despite evidence for decreased mPFC activation following successful first‐line treatment, the effects of alternative treatments on amygdala activation or volume remain unclear (Manthey et al., 2021; Quidé et al., 2012). Other regions, such as the thalamus, the precuneus, and the occipital cortex, have also been inconsistently associated with PTSD and/or trauma exposure (Patel et al., 2012; Quidé et al., 2021; Sartory et al., 2013).

Both psychological and pharmacological approaches have been recommended for treating PTSD. First‐line psychological interventions for PTSD include trauma‐focused cognitive behavioral therapy, although other recommended treatments, including eye movement desensitization and reprocessing, cognitive processing therapy, or prolonged exposure therapy, are also widely used (Asmundson et al., 2019; Bisson & Olff, 2021), whereas antidepressants such as selective serotonin reuptake inhibitors (SSRIs), tricyclic antidepressants, or monoamine oxidase inhibitors have been recommended as first‐line pharmacological options (Albucher & Liberzon, 2002). A meta‐analysis of pharmacological treatments indicates that sertraline, fluoxetine, paroxetine, prazosin, venlafaxine, quetiapine, and risperidone have a small positive effect with no evidence of superiority for one intervention over another (Hoskins et al., 2021). The use of SSRIs has been linked with both changes in brain function and morphology in PTSD (Quidé et al., 2012): Fluoxetine was found to normalize hyperactivity of the cerebellum, precuneus, and supplementary motor cortex (Fernandez et al., 2001), whereas paroxetine was associated with increased hippocampal volume (Vermetten et al., 2003) and increased frontal (orbitofrontal cortex, anterior cingulate cortex) function during trauma versus neutral script presentations (Fani et al., 2011).

Evidence for the efficacy of current antidepressant approaches is limited, with about one third of PTSD patients still meeting criteria for PTSD after treatment (Hoskins et al., 2021; Sijbrandij et al., 2015). As such, alternative approaches have been proposed (De Kleine et al., 2013). A popular approach is to use intranasal oxytocin: Oxytocin receptors are particularly present in key regions involved in PTSD, including the amygdala, hippocampus, parahippocampal gyrus, anterior cingulate cortex, hypothalamus, or olfactory bulb (Boccia et al., 2013). Intranasal oxytocin administration in non‐trauma‐exposed healthy individuals modulates the activity of these brain regions and associated networks, reaching to the insula, nucleus accumbens, or the dorsomedial prefrontal cortex (Feng et al., 2015; Olff et al., 2010; Rilling et al., 2014). By modulating the activity of these brain regions and networks, oxytocin can assist with fear regulation and reward processing (Harari‐Dahan & Bernstein, 2014) by enhancing motivational salience and increasing the desire for social interaction (Yatzkar & Klein, 2010). Similarly, cannabinoids have also been proposed to treat PTSD (Mayo et al., 2022). The endocannabinoid system is essential in fear extinction (Hill et al., 2018), learning, and memory (Ruehle et al., 2012). Low dose of delta‐9‐tetrahydrocannabinol (THC), the psychoactive constituent of cannabis, significantly reduces amygdala reactivity to social cues of threat and fear inhibition tasks (Rabinak et al., 2014, 2020). More recently, ketamine has been proposed as a new option to treat chronic PTSD (Ragnhildstveit et al., 2023). This non‐competitive antagonist of the *N*‐methyl‐d‐aspartate glutamate receptor can reverse the effects of trauma and chronic stress on the amygdala, hippocampus, and prefrontal cortex (Duman et al., 2012), thus improving mood and fear learning by promoting neurogenesis, synaptogenesis, and cell proliferation (Clarke et al., 2017; Duman et al., 2012). Finally, the use of psychedelics, especially 3,4‐methylenedioxymethamphetamine (MDMA), has been used to augment the beneficial effects of psychotherapies in PTSD: MDMA is thought to reduce the fear response associated with re‐experiencing traumatic memories, to facilitate tolerable processing of traumatic content (memory reconsolidation, fear extinction) in patients with PTSD (Mithoefer et al., 2011). These effects are thought to target the functional connectivity between the amygdala and hippocampus in healthy volunteers (Carhart‐Harris et al., 2015) and in PTSD patients (Singleton et al., 2022).

Other pharmacological agents have been proposed to prevent the development of PTSD, early following exposure to traumatic events. Administration of hydrocortisone, a synthetic form of cortisol, early following trauma exposure has a large effect in reducing the risk of PTSD (Sijbrandij et al., 2015). Glucocorticoid receptors are widely expressed in the hippocampus, the amygdala, and the prefrontal cortex, to regulate the hypothalamic–pituitary–adrenal axis activity (Mcwen, 2017; Smith & Vale, 2006). Glucocorticoid administration may offer critical therapeutic advances (De Quervain et al., 2003), by reducing the retrieval of traumatic memories and promote extinction and inhibitory fear learning (De Quervain et al., 2009; Sijbrandij et al., 2015), and can enhance the effects of prolonged exposure therapy (Yehuda et al., 2015). Similarly to the use of glucocorticoids, the beta‐adrenergic blocker propranolol is used to prevent the development of PTSD by blocking the consolidation of fearful memories via the dorsal mPFC (dmPFC) and improving the learning of a safe context by the hippocampus (Kroes et al., 2016). Although early evidence indicates that propranolol use is associated with decreased symptoms and increased activation in the right anterior cingulate cortex in response to fearful versus happy faces (Mahabir et al., 2015), the overall efficacy of propranolol in preventing PTSD is unclear (Sijbrandij et al., 2015).

Although evidence indicates that psychotherapy may upregulate the mPFC, leading to symptom reduction in PTSD patients (Manthey et al., 2021), the neural correlates underlying successful alternative pharmacological intervention in PTSD remain unclear. Understanding the effects of these new treatment options on brain function is critical to extend existing models in order to consider the translational value of selected drugs or drug receptors into clinical therapy (Trist et al., 2014) and to provide the best intervention options to people suffering from PTSD. The current study reviews the evidence resulting from clinical trials evaluating the changes in brain function following the use of these alternative pharmacological agents in PTSD patients.

## 2 MATERIALS AND METHODS

### 2.1 Search strategy

**FIGURE 1:**
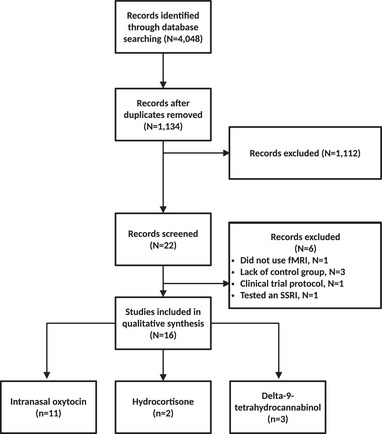
Preferred reporting items for systematic reviews and meta‐analyses (PRISMA) flow diagram.

Adhering to the Preferred Reporting Items for Systematic Reviews and Meta‐Analyses (PRISMA) 2020 statement (Page et al., 2021), the PubMed, Scopus, and Web of Science databases were searched for studies published before September 2022. The search was limited to clinical trials estimating the effects of alternative pharmacological agents other than first‐line pharmacological treatments (e.g., SSRIs), on resting‐state or task‐related functional magnetic resonance imaging (fMRI) outcomes. The search was performed on PubMed using the following MESH terms: stress disorders and posttraumatic and magnetic resonance imaging. The search was then restricted to Clinical Trials (search filters “article type: Clinical Trial”). Search on Web of Science and Scopus using the search terms *MRI, fMRI, neuroimaging, magnetic resonance imaging* crossed with *therapeutic, treatment, intervention* and again fully crossed with the terms *posttraumatic stress disorder, PTSD*. The study selection processes are outlined in the flow diagram (see Figure 1).

### 2.2 Study selection

Inclusion criteria included original articles on adult patients with PTSD, diagnosed based on either the criteria from the Diagnostic and Statistical Manual of Mental Disorders fourth or fifth editions (DSM‐IV, DSM‐IV‐TR, and DSM‐5) (APA, 1994, 2000, 2013) or the International Statistical Classification of Diseases and Related Health Problems (ICD‐10) (World Health Organization, 1992). Studies including a healthy control (HC) group and/or a group of TEC who did not develop PTSD following trauma were considered to determine both the unspecific (irrespective of the group) and specific effects of the studied pharmacological agents in PTSD patients relative to their control groups. Studies that were not clinical trials, studies that did not report fMRI as their outcome measures, studies with incomplete reporting, studies comparing two pharmacological interventions, studies not including an HC or TEC comparison groups, and studies published in other languages than English were excluded. Clinical trials allowing concomitant antidepressant use were excluded. Two reviewers independently screened the identified abstracts. Extracted data included the year of publication, sex, age, diagnosing tool, type of fMRI (resting state of task‐related fMRI), and the name of the task for task‐related fMRI studies.

### 2.3 Data extraction

Study characteristics were extracted, including sample size, diagnostic tools, type of treatment, and type of brain imaging (see Table 1). In addition, the relevant results from each study, including the main effects of the treatment of interest, the main effects of groups, and the effects of the group‐by‐treatment interactions on brain function, were extracted and are reported in Table 2. Other results within each study were not reported here if they were not related to the aims of the present study.

**TABLE 1:** Details of the included studies.

Study	Sample size of patients (female)	Sample size of control group (female) Type of control group	Diagnostic tools	Type of treatment versus control (sham, placebo, other drug)	Type of fMRI
**Intranasal oxytocin**					
Nawjin et al. (2015)	35 (14)	37 (18)—TEC	CAPS‐MINI‐SCID	40IU versus placebo	Monetary incentive delay
Koch et al. (2016)	37 (16)	40 (20)—TEC	CAPS‐MINI‐SCID	40IU versus placebo	Resting‐state and emotional face‐matching, monetary and social incentive delay, reward tasks
Frijling et al. (2016)	24 (15)	20 (11)—HC	TSQ‐PDI‐CAPS‐MINI‐ETI	40IU versus placebo	Resting‐state and Script‐driven imagery
Frijling et al. (2016)	23 (14)	18 (10)—HC	TSQ‐CAPS‐MINI	40IU versus placebo	Emotional face‐matching task
Nawijn et al. (2015)	35 (All)	37 (All)—HC	CAPS‐MINI‐SCID	40IU versus placebo	Social incentive delay task
Koch et al. (2018)	36 (15)	40 (20)—TEC	CAPS‐MINI‐SCID	40IU versus placebo	Distraction task
Flanagan et al. (2018)	16 (7)	18 (11)—TEC	CTQ‐MINI‐SCID‐CAPS	24IU versus placebo	Working memory task
Flanagan et al. (2019)	17 (8)	16 (10)‐TEC	CAPS‐SCID‐CTQ‐STAI	24IU versus placebo	Facial affect recognition
Sippel et al. (2021)	18 (12)	16 (9)—TEC	CTQ‐CAPS‐SCID‐PDS	24IU versus placebo	Facial affect recognition task
Crum et al. (2021)	15 (7)	16 (9)—TEC	CAPS‐PTDS‐MINI‐CTQ	24IU versus placebo	Resting‐state
**Hydrocortisone**					
Metz et al. (2018)	20 (All)	40 (All)—HC	SCID‐PDS‐r‐CTQ‐ELS	10 mg	Resting‐state fMRI
Metz et al. (2019)	20 (All)	40 (All)—HC	SCID‐PDS‐r‐ CTQ	10 mg	Autobiographical memory test
**Delta‐9‐tetrahydrocannabinol (THC)**					
Rabinak et al. (2020)	19 (14)	52 (22)—HC, TEC	CAPS‐LEC‐5	7.5 mg versus placebo	Threat processing task
Pacitto et al. (2022)	22 (15)	30 (14)—TEC	CAPS‐LEC‐5	7.5 mg versus placebo	Emotion regulation task
Zabik et al. (2023)	19 (13)	52 (21)—HC, TEC	CAPS‐LEC‐5	7.5 mg versus placebo	Fear conditioning and extinction paradigm

Abbreviations: CAPS, clinician‐administered PTSD scale; CTQ, child trauma questionnaire; ELS, early life stress; ETI, early trauma inventory; HAM‐D, Hamilton depression rating scale; HC, healthy controls; IU, international unit; LEC‐5, the life events checklist for DSM‐5; MADRS, Montgomery–Åsberg depression rating scale; MINI, mini‐international neuropsychiatric interview; MG, milligrams; PDI, peritraumatic distress inventory; PDS‐r, posttraumatic diagnostic scale; SCID, structured clinical interview for DSM; STAI, state‐trait anxiety inventory; TEC, trauma‐exposed controls; TSQ, trauma screening questionnaire.

**TABLE 2:** Effects of interest extracted from the included publications.

Study	Effects of treatment of interest	Effects of group	Interaction group x treatment
**Intranasal oxytocin**			
a. Resting‐state fMRI studies			
Crum et al. (2021)	↓ Connectivity between DAN and VAN	–	–
Koch et al. (2016)	–	–	**Males** **Oxytocin versus placebo (PTSD)**: ↑ right centromedial amygdala and left vmPFC connectivity **Males PTSD versus TEC (placebo)**: ↓ right centromedial amygdala and left vmPFC connectivity **Females** **Oxytocin versus placebo (PTSD)**: ↓ right basolateral to bilateral dorsal anterior cingulate cortex **PTSD versus TEC (placebo)**: ↑ right basolateral to bilateral dorsal anterior cingulate cortex
Frijling et al. (2016)	↑ Connectivity between the amygdala and the insula ↓ connectivity between the amygdala and the ventro‐lateralPFC	–	**Oxytocin: Trauma versus neutral script** ↓ connectivity between the amygdala and the left vmPFC **Placebo: Trauma versus neutral script** ↑ connectivity between the amygdala and the left vmPFC
b. Working memory task			
Flanagan et al. (2018)	–	–	**PTSD versus TEC (oxytocin)** ↑ connectivity between the dorsolateral prefrontal cortex and anterior cingulate increased in the 2‐back condition
c. Emotional processing			
Koch et al. (2016)	–	‐	**Oxytocin versus placebo** **PTSD**: ↓ amygdala reactivity **HC**: ↑ amygdala reactivity
Frijling et al. (2016)	–	–	**Oxytocin versus placebo (PTSD)** **Fearful faces**: ↑ right basolateral amygdala reactivity **Neutral faces**: ↑ left basolateral amygdala reactivity in women (not men)
Sippel et al. (2021)	–	–	**Oxytocin versus p lacebo** **PTSD**: ↓ left amygdala and left anterior insula connectivity for females Left amygdala‐ right anterior insula connectivity ↓ for females and ↑ for males Right amygdala–right anterior insula connectivity for ↓ males and for ↑ females
Flanagan et al. (2019)	–	–	**Oxytocin versus placebo** **PTSD**: ROI: ↑ right amygdala and ↓ left amygdala to fearful faces; exploratory whole‐brain analyses: right occipital gyrus, right precentral/cingulate gyrus, left insula/inferior frontal gyrus **TEC: ROI**: ↑ left amygdala to fearful faces; exploratory whole‐brain analyses: bilateral fusiform gyrus
d. Social incentive delay task			
Nawijn et al. (2017)			**Oxytocin versus placebo** **PTSD**: ↑ right putamen activation and left insula in response to reward feedback **HC**: ↓ right putamen activation and left insula in response to reward
e. Monetary incentive delay task			
Nawijn et al. (2016)	↑ Left and right putamen, dorsal anterior cingulate cortex, and insula responses during reward and loss anticipation		
f. Distraction task			
Koch et al. (2016)	↑ Left thalamus activation		**Oxytocin** PTSD: ↑ connectivity between the left thalamus and the amygdala (males + females) TEC: ↑ connectivity between the left thalamus and the amygdala in males (not females)
**Hydrocortisone**			
Metz et al. (2018)	–	–	–
Metz et al. (2019)	–	–	–
**Delta‐9‐tetrahydrocannabinol (THC)**			
a. Threat processing task			
Rabinak et al. (2020)	–	–	**THC versus placebo** **PTSD**: ↓ amygdala activity and ↑ mPFC and ↓ amygdalostriatal and ↑ mPFC/rACC response to threat **TEC**: ↑ basolateral amygdala **HC**: ↓ amygdalostriatal response to non‐threat
b. Emotion regulation task			
Pacitto et al. (2022)	–	–	**THC versus placebo** **PTSD**: ↑ dmPFC activation during exposure to neutral images and ↑ cerebellar and posterior cingulate cortex (PCC)/precuneus activation during reappraisal ↑ angular gyrus
c. Fear conditioning and extinction paradigm			
Zabik et al. (2023)	–	–	**THC versus placebo** **PTSD**: ↑ vmPFC and amygdala **HC**: ↑ vmPFC

Abbreviations: HC, healthy controls; PFC, prefrontal cortex; PTSD, posttraumatic stress disorder; ROI, region‐of‐interest; TEC, trauma‐exposed controls; THC, delta‐9‐tetrahydrocannabinol; vmPFC, ventromedial prefrontal cortex.

### 2.4 Risk of bias assessment

Two authors (SL and AY) independently assessed the risks of bias using the Cochrane Collaboration's Tool for Assessing Risk of Bias in Randomized Trials (RoB 2.0) (Sterne et al., 2019). In addition to provide an overall bias estimation, this tool assesses five domains of potential bias for each individual study (selection of the reported result, measurement of the outcome, missing outcome data, deviation from intended interventions and randomization process) and ranks them as “low,” with “some concern” or “high.” In the event of discrepancy in bias assessment between reviewers, consensus was achieved through discussion.

## 3 RESULTS

A total of 4048 articles were identified. After removing duplicates, abstracts, and titles, 1134 papers were screened, from which 1112 articles were excluded. Twenty‐two articles were screened, with six records excluded because they did not satisfy the inclusion criteria (lack of control group, *N* = 3), tested the effects of an SSRI (paroxetine; *N* = 1), did not use fMRI (*N* = 1), and referred to a clinical trial protocol (*N* = 1). Sixteen articles were included in the review (see Figure 1 and Table 1); among them, 11 used intranasal oxytocin (3 resting‐state fMRI, 8 task‐related fMRI, from 4 distinct datasets), 2 used hydrocortisone (1 resting‐state fMRI, 1 task‐related fMRI, from one dataset), and 3 task‐related fMRI used THC (from one dataset). Effects of interventions, groups, and their interactions on brain function are reported in Table 2.

### 3.1 Oxytocin

#### 3.1.1 Resting‐state fMRI

Koch et al. 2016 used resting‐state fMRI to investigate the effects of intranasal oxytocin on the functional connectivity of the basolateral and centromedial amygdala in police officers with (*n* = 37) and without PTSD (*n* = 40) (Koch et al., 2016). Following the administration of oxytocin, connectivity between the right centromedial amygdala and the left ventromedial prefrontal cortex (vmPFC) was normalized (enhanced) in male PTSD cases relative to placebo, reaching a similar level than the TEC group after oxytocin administration. Connectivity between these two regions was reduced in PTSD males compared to TEC males after placebo administration. There were effects of intervention or group on connectivity between the left vmPFC and the right centromedial amygdala in females. However, PTSD females showed reduced connectivity between the right basolateral to bilateral dorsal anterior cingulate cortex following oxytocin administration, compared to placebo (Koch et al., 2016), whereas placebo was associated with enhanced connectivity between these regions in PTSD females, compared to TEC females. Compared to TEC (*n* = 16), childhood trauma survivors with PTSD (*n* = 15) showed reduced the strength of resting state connectivity between the dorsal (DAN; made of the precuneus, occipital cortex, parietal cortices, middle and superior frontal gyri, temporo‐occipital gyrus, and precentral gyrus) and ventral attentional networks (made of the supplemental motor area/superior frontal gyrus, bilateral superior and middle temporal gyrus, and bilateral inferior frontal gyrus) (Crum et al., 2021). This pattern of connectivity was found increased after oxytocin administration in both groups.

#### 3.1.2 Emotional processing

Four studies have investigated the effects of intranasal oxytocin administration while performing an emotional face‐matching task. In their cohort of police officers, Koch et al. examined the effects of oxytocin on amygdala reactivity toward emotional stimuli (Koch et al., 2016). Compared to placebo (*n* = 40), oxytocin administration (*n* = 38) was associated with decreased amygdala reactivity in PTSD patients and enhanced amygdala reactivity in HCs. The intervention moderated the relationship between pre‐intervention anxiety scores and amygdala reactivity: This association between anxiety and amygdala reactivity was dampened after oxytocin administration, whereas it increased in the placebo group. In acute civilian PTSD patients, a single intranasal oxytocin administration (*n* = 23) showed enhanced right basolateral amygdala reactivity to fearful faces compared to the placebo group (*n* = 18). Results showed a significant intervention‐by‐sex interaction on amygdala reactivity to neutral faces, with women showing enhanced left amygdala reactivity to fearful stimuli after oxytocin administration (Frijling et al., 2016).

In childhood trauma survivors with (*n* = 16) or without PTSD (*n* = 18), oxytocin administration was associated with reduced left amygdala–left anterior insula functional connectivity in response to fearful face presentation in females but not males, as well as reduced left amygdala–right anterior insula connectivity among females that was increased in males, and reduced right amygdala–right anterior insula connectivity in males that was increased in females (Sippel et al., 2021). Finally, in the same sample, there was no effect of oxytocin on amygdala function, relative to placebo in the PTSD group. However, changes in left amygdala response to fearful face presentation were negatively associated with childhood trauma severity in individuals with PTSD (*n* = 17), whereas increased following oxytocin administration in the control group (*n* = 16). In addition, exploratory analyses outside of the amygdala indicated that the PTSD group showed larger patterns of activation in response to fearful faces in the right occipital gyrus, the right precentral/anterior cingulate gyrus, and the left insula‐inferior frontal gyrus (Flanagan et al., 2019).

#### 3.1.3 Social incentive delay task

Only one study investigated brain response to oxytocin when performing a social incentive delay task in police officers (Nawijn et al., 2017). Compared to placebo (*n* = 36), intranasal oxytocin administration (*n* = 35) was associated with increased activation in the right putamen and left insula in response to reward feedback in the PTSD group, whereas decreased activation in these regions was evident in the control group.

#### 3.1.4 Trauma‐related script (script‐driven imagery)

In a study of 44 civilians with acute PTSD (*n* = 24 received oxytocin and *n* = 20 received a placebo) recruited early following trauma exposure (scanning session within 11 days post trauma) (Frijling et al., 2016), functional connectivity at rest between the (whole) amygdala and the left ventrolateral prefrontal cortex was weaker in response to a previously presented trauma‐related script compared to a neutral script following oxytocin administration, whereas this association was stronger in the placebo group. Irrespective of the script condition, the administration of oxytocin increased the strength of functional connectivity between the amygdala and the insula and decreased the strength of connectivity between the amygdala and the vmPFC (Frijling et al., 2016).

#### 3.1.5 Working memory

Only one study investigated the effects of oxytocin on brain function during the performance of an *n*‐back working memory task in people with childhood trauma‐related PTSD (*n* = 16) compared to childhood trauma TEC (*n* = 18) (Flanagan et al., 2018). Compared to placebo, in the context of improved performance at the most difficult condition (2‐back), connectivity between the dmPFC and anterior cingulate cortex increased in the 2‐back condition among individuals with PTSD using oxytocin (relative to TEC) (Flanagan et al., 2018).

#### 3.1.6 Monetary incentive delay task

Only one study evaluated the effects of oxytocin on brain function during the performance of a monetary incentive delay task in police officers with (*n* = 35) or without PTSD (*n* = 37) (Nawijn et al., 2016). Compared to placebo, oxytocin administration enhanced neural responses during reward and loss anticipation in both PTSD patients and controls in the left and right putamen, dorsal anterior cingulate cortex, and insula. Additionally, oxytocin relieved these anhedonia‐related neural reward deficits, suggesting that oxytocin may increase motivation and reward sensitivity.

#### 3.1.7 Distraction task

The only study investigating the impact of oxytocin administration on brain response during the performance of a distraction task (Koch et al., 2019) reported that former police officers showed enhanced left thalamus activation during the task after oxytocin administration compared to placebo, independently of being diagnosed (*n* = 37) or not with PTSD (*n* = 40). In addition, oxytocin administration was associated with increased left thalamus activity and task‐related functional connectivity with the amygdala in all PTSD patients and in male, but not female, TEC.

In summary, when processing emotional/traumatic stimuli, the administration of oxytocin was found to reduce amygdala reactivity, normalize mPFC–amygdala and amygdala–insula connectivities, and reduce the connectivity between ventral (affective) and dorsal (cognitive) attentional networks in PTSD. During the performance of (nonemotional) cognitive tasks, oxytocin administration was associated with increased activation and functional connectivity among regions critical for the cognitive processes studied: dorsolateral PFC and anterior cingulate cortex during the performance of a working memory task, putamen, dorsal anterior cingulate cortex, and insula during the performance of a reward, and thalamus during the performance of an attentional task. Importantly, sex differences have been observed.

## 4 Hydrocortisone

One group studied the effects of hydrocortisone on the brain function of females with PTSD (*n* = 20), borderline personality disorder (*n* = 18), and HCs (*n* = 40). There were no effects of hydrocortisone on patterns of resting‐state functional connectivity (Metz et al., 2019) or brain function during the performance of an autobiographical memory task (Metz et al., 2019).

## 5 Delta‐9‐tetrahydrocannabinol (THC)

Three studies from the same group, with sub‐samples of the same dataset, indicate that THC administration [HC (*n* = 25), TEC (*n* = 27), and PTSD (*n* = 19)] was associated with reduced amygdala reactivity and increased activation of the mPFC, along with amygdala‐mPFC functional connectivity during threat in adults with PTSD (Rabinak et al., 2020). Relative to placebo (*n* = 26), administration of THC (*n* = 25) was associated with no overall changes in brain function during the performance of an emotion regulation task (independently of the condition) in people with PTSD (*n* = 21) compared to TEC (*n* = 30) (Pacitto et al., 2022). However, in the PTSD group only, THC administration (*n* = 10), compared to placebo (*n* = 11), was associated with increased activation of the cerebellum when participants were asked to passively process neutral images (maintain neutral condition) only. Finally, while performing a fear conditioning and extinction paradigm, THC administration was associated with greater vmPFC activation in people with PTSD (*n* = 9) compared to TEC (*n* = 12), while greater activation of the hippocampus was evident during late (relative to early) extinction recall in the right hippocampus, independently of group (both PTSD and TEC) (Zabik et al., 2023). Relative to placebo, THC administration was also associated with greater left amygdala activation during early presentations of the extinguished conditioned stimulus (relative to the unpaired conditioned and unconditioned stimuli) in PTSD.

## 6 Bias assessment

**FIGURE 2:**
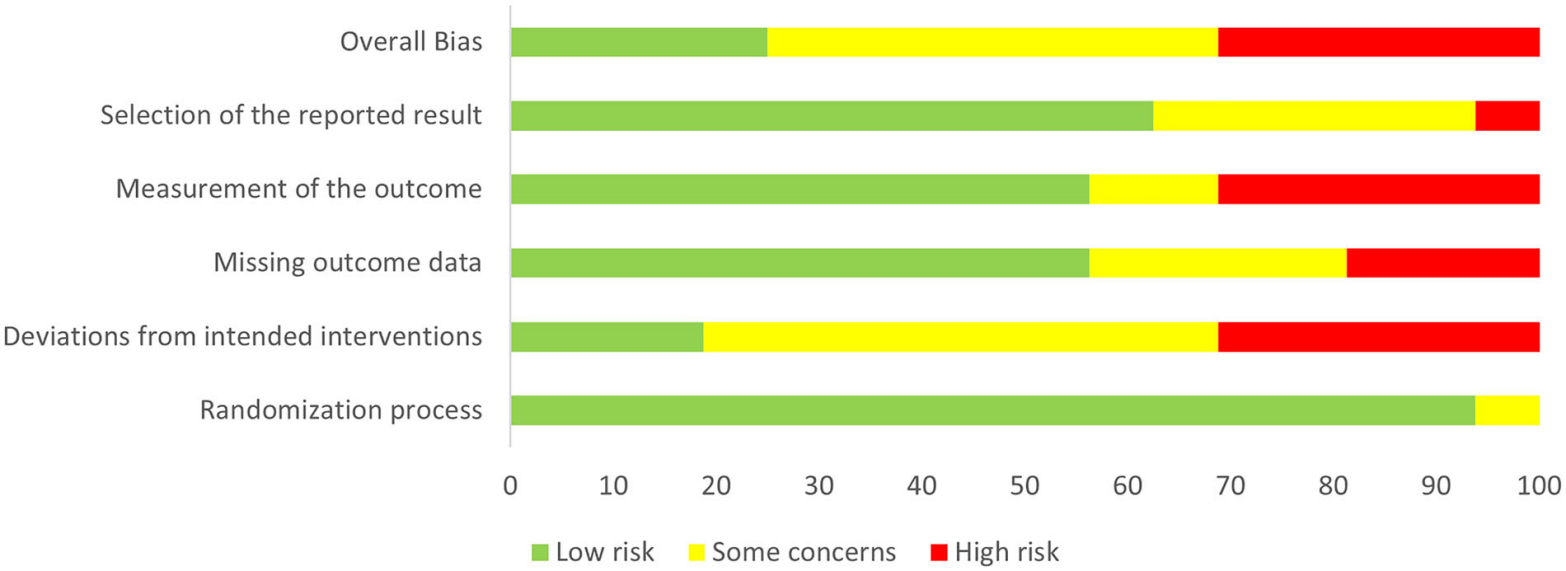
Bias assessment of the included studies.

The overall quality assessment of the selected articles indicates that 25% of studies show low risk, 43.8% of studies included show some concerns, and 31.3% being considered at high risk (see Figure 2). Notably, the deviations from intended interventions were estimated being at 18.8% with low risk, 50% with some concerns, and 31.3% at high risk, whereas the selection of reported results was estimated to be at low risk for 62.5% of the included studies (31.3.2% with some concerns and 6.3% at high risk), and the measurement of the outcome and the missing outcome data were at low risk in 56.3% (12.5% with some concerns, 31.3% at high risk for the measurement of the outcome, 25% with some concerns, and 18.8% at high risk for the missing outcome). Finally, the randomization process was estimated being at low risk of bias in 92.8% of the included studies (6.3% with some concerns).

## 7 DISCUSSION

The present systematic review identified 16 clinical trials investigating the impacts of alternative pharmacological agents (oxytocin, hydrocortisone, and THC) on brain function in PTSD. Although these studies used various imaging paradigms (resting‐state fMRI and task‐related fMRI paradigms), results mostly converge toward similar functional changes in key regions involved in PTSD, including the amygdala, the medial prefrontal, dorsolateral prefrontal, anterior cingulate cortex, and parietal cortex. Sex‐specific effects were also reported, especially following the administration of oxytocin.

### 7.1 Functional brain changes after oxytocin administration

At rest, oxytocin administration was associated with changes in functional connectivity between frontal (e.g., vmPFC) and limbic and affective regions (e.g., amygdala). Normalization (increase) of the connectivity between the vmPFC and the amygdala, especially its centromedial nucleus, was evident in male, but not female, patients with PTSD (Koch et al., 2016). This was further evident following exposure to a trauma‐related script (Frijling et al., 2016). Based on the amygdalo‐centric model of PTSD (Rauch et al., 2006), this increased connectivity may reflect increased top–down prefrontal control over the fear response. Interestingly, oxytocin administration was also associated with reduced connectivity strength between dorsal (mostly cognitive) and ventral (mostly affective) attentional networks (Crum et al., 2021), suggesting that this pharmacological agent may help reduce focus on affective inner thoughts.

During the performance of cognitive tasks, oxytocin administration was associated with changes in activation in brain regions critical for the tasks studied. During the performance of a working memory task, oxytocin administration was associated with increased connectivity between the left dorsolateral prefrontal and anterior cingulate cortices, with greater effects in people diagnosed with PTSD compared to TEC (Flanagan et al., 2018). The direction of effect observed between these two regions was not consistent with normalization of the aberrant patterns of connectivity in PTSD, suggesting that oxytocin‐related enhanced recruitment of this circuit may reflect some compensatory mechanisms to adequately perform the task and may not necessarily reflect increased cognitive efficiency or performance monitoring. During the performance of emotional tasks, results were inconsistent: Administration of oxytocin (compared to placebo) was associated with decreased amygdala activity in a group of police officers (Koch et al., 2016), while associated with increased reactivity of the right basolateral amygdala in a group of acute civilian PTSD patients (Frijling et al., 2016). This discrepancy may be related to the fact that police officers may have been repeatedly exposed to more stressful, if not traumatic events, than the group of acute PTSD patients. Sensitization of the stress system to repeated stress exposure might impact the baseline levels of amygdala reactivity and influence the way oxytocin modulates it. This explanation remains speculative, and future studies including groups of PTSD patients with different levels of basal stress and trauma exposure may help understanding how oxytocin administration may differently influence emotional processing. This is critical to provide the most efficient intervention to people suffering from PTSD.

Other studies have reported enhanced activation in striatal regions following oxytocin administration when performing rewarding tasks, such as social or monetary incentive delay task (Nawijn et al., 2016, 2017). Although oxytocin was associated with increased putamen, anterior cingulate and insular activation independently of the group during reward and loss anticipation, increased putamen and insular activations were specifically observed in PTSD, and decreased in HCs, when social reward feedback was sought after. These findings may indicate that reward evaluation in PTSD may be facilitated by oxytocin administration enhancing motivational processing. However, the impact of oxytocin on brain function during these tasks is highly sensitive to sex, baseline levels of oxytocin system function, and potentially the type of traumatic event and chronicity of PTSD (Olff et al., 2013). Finally, when performing a distraction task (Koch et al., 2019), oxytocin administration was found to modulate thalamic activation, and connectivity with the amygdala, suggesting that oxytocin administration can enhance neural emotional control in PTSD. As suggested by the authors, intranasal oxytocin administration could be used as an enhancer for the changes in brain function produced by first‐line psychological approach for PTSD, such as cognitive‐behavioral therapy. This will need to be further tested in future trials. If these effects are confirmed, the association of oxytocin and CBT may provide important new avenues for the treatment of PTSD.

### 7.2 Functional changes after hydrocortisone administration

Effects of other pharmacological agents in PTSD are less evident. For instance, hydrocortisone administration was not associated with any changes in brain function, either at rest or during the performance of an autobiographical memory task (Metz et al., 2019). The relatively small sample size of these studies may have limited the power to detect more subtle effects. It may also be the case that hydrocortisone may have long‐term effects, not captured in the present trials. Importantly, only two clinical trials using hydrocortisone have been published and met the inclusion criteria for the present systematic review. This strongly limits our understanding of the benefits of hydrocortisone as an alternative therapeutic approach or for PTSD. It would be important to replicate and confirm the lack of effects in larger populations, potentially in groups of patients exposed to different types of trauma. Larger samples allowing the investigation of sex‐specific effects of hydrocortisone on brain function are also warranted, as sex‐specific effects of PTSD on the stress system, especially on cortisol production and regulation, are now well established (Meewisse et al., 2007).

### 7.3 Functional changes after delta‐9‐tetrahydrocannabinol (THC) administration

From the limited number of studies included in this systematic review, the administration of THC was associated with reduced amygdala reactivity to threat and enhanced cortico‐limbic activation during extinction learning (Rabinak et al., 2020; Zabik et al., 2023). According to the authors, these results indicate that the administration of an acute low dose of THC may help facilitate successful emotional regulation and self‐referential processing (Pacitto et al., 2022). In addition, stronger top–down regulation from the vmPFC toward the amygdala during extinction learning in people with PTSD, relative to TEC (Zabik et al., 2023), indicates that THC administration may enhance successful fear reduction and successful extinction learning (Milad & Quirk, 2002). As noted, the limited number of studies, using the same dataset, prevents a clear understanding of the therapeutic effects of THC in PTSD. However, early evidence suggests that a low dose of THC may contribute to the fear of extinction if administered early posttrauma exposure. The effects of higher doses of THC or combinations of THC and varied levels of cannabidiol remain unclear and understudied. Future clinical trials are needed to better determine if THC, in conjunction with other cannabinoids or alone, at low or higher dose, in combination with psychotherapies or alone, may be an ideal approach to prevent the development of PTSD.

### 7.4 Limitations

This systematic review has several limitations. First, the present systematic review only focused on fMRI studies, excluding alternative methods to investigate changes in brain function (e.g., arterial spin labeling, positron emission tomography, single‐photon emission computerized tomography, and electroencephalography) or morphology. Although out of the scope of this systematic review, we cannot completely rule out that the use of some of these alternative pharmacological agents may have induced subtle morphological changes during the course of the presented clinical trials. In addition, the interpretations of the findings mentioned above are limited by the number of studies included and the heterogeneous research methods used, including the types of tasks, as well as the way drugs were administered (e.g., intranasal vs. oral administration). Other recent studies using ketamine (e.g., [Carhart‐Harris et al., 2014; Norbury et al., 2021], psychedelics [Singleton et al., 2022], propranolol [Mahabir et al., 2015], tolcapone [an inhibitor of the degradation of dopamine by catechol‐O‐methyl transferase] [Westphal et al., 2021] or aprepitant [a neurokini‐1 receptor antagonist] [Kwako et al., 2015]) were not included as they did not compared patients with PTSD to groups of TEC or HCs. Furthermore, differences in symptoms severity, trauma types, and age of trauma onset were not considered. The results reported here suggest strong sex‐specific effects of oxytocin, and not all included studies attempted to investigate these effects. Finally, the included studies were, in general, of small sample sizes, limiting their replicability and generalizability.

## 8 CONCLUSIONS

Despite the heterogeneity of methods and pharmacological agents used, the present systematic review of clinical trials has identified preliminary evidence for the normalization of brain function following the administration of pharmacological agents. Oxytocin and THC administration were overall associated with normalized amygdala function and amygdala–mPFC connectivity, as well as critical regions for cognitive processes studied (e.g., dmPFC in working memory, putamen in reward, hippocampus in fear condition, and extinction), whereas hydrocortisone was associated with no significant functional brain changes. Interpretation of the results from this systematic review needs to be cautious due to the limited number of clinical trials of small sample sizes included. Further clinical trials are necessary to better understand the neurobiological mechanisms and effects of these and other alternative pharmacological agents on brain function and morphology, alone or in combination with other approaches (e.g., psychotherapies), to benefit people suffering from PTSD.

## AUTHOR CONTRIBUTIONS

**Shahab Lotfinia**: Conceptualization; data curation; formal analysis; investigation; methodology; project administration; resources and writing—original draft. **Amin Afshar**: Data curation; formal analysis; investigation; resources; writing—review and editing. **Aram Yaseri**: Data curation; formal analysis; investigation; resources; writing—review and editing. **Miranda Olff**: Methodology; project administration; supervision; writing—review and editing. **Yann Quidé**: Investigation; methodology; supervision; writing—review and editing.

## CONFLICT OF INTEREST STATEMENT

The authors declare that they have no conflicts of interest.

### FUNDING INFORMATION

No funding was received.

### PEER REVIEW

The peer review history for this article is available at https://publons.com/publon/10.1002/brb3.3292.

## Data Availability

The data that support the findings of this study are available from the corresponding author upon reasonable request.
